# Expanding climate policy adoption improves national mitigation efforts

**DOI:** 10.1038/s44168-023-00043-8

**Published:** 2023-06-09

**Authors:** Leonardo Nascimento, Niklas Höhne

**Affiliations:** 1 Environmental System Analysis, Wageningen University and Research, Wageningen, The Netherlands; 2 NewClimate Institute, Cologne, Germany

**Keywords:** Climate-change mitigation, Projection and prediction

## Abstract

To identify means to improve mitigation efforts, we investigated whether the number of climate policies is associated with emission projections up to 2030 and compared policies’ prevalence across country groups. We find that larger and more comprehensive policy portfolios are conducive to emission reductions, regardless of whether absolute emissions increase or already decline. However, country groups have distinct entry points to expand climate policy. Countries with fast-increasing emissions have significantly fewer policies overall but policies are especially missing in energy-demand sectors, such as buildings and transport. Countries with stalling emissions lack climate strategies and other cross-sectoral policies. This suggests the need for better coordination of mitigation efforts across sectors. In all country groups that fail to reduce emissions, policies to reduce energy and material demand are also substantially fewer. Despite the collective increase of policies in force, countries can still expand climate policy to use the full breadth of mitigation options available.

## Introduction

Climate change results in unprecedented and rapid changes in the Earth’s systems^1^. These changes are expected to reach devastating levels if countries do not reduce their greenhouse gas emissions, hereafter emissions, to zero and collectively limit the end-of-century temperature increase to 1.5 °C^2^. To mitigate climate change, countries adopt and implement several policies. These policies include those with explicit mitigation objectives, such as climate strategies; energy policies, that help to decarbonise energy supply and/or reduce demand; and policies that introduce low-emissions practices to non-energy sectors^3^. However, policies to date have been insufficient to curb historical global emissions^4,5^.

Although policies adopted so far fail to secure emissions reductions in line with global decarbonisation, their number increased in the past few decades^6^. Distinct statistical analyses provide evidence of policies’ aggregated effect^7,8^ or of their relationship to country characteristics^9,10^. These analyses show that collectively climate policies led to a net-positive effect and contributed to a reduction of historical emissions^11^. This shows that increasing the number of climate policies improved climate change mitigation efforts. However, the pathways leading to impact require further investigation^12^.

Additionally, evaluating emission projections resulting from adopted policies constitutes an important tool to assess whether current efforts are sufficient to limit temperature increase^13^. In 2021, adopted policies resulted in lower emission projections up to 2030 than estimated in 2015^14^. However, global emissions are projected to remain on an upwards trend^15^. This suggests that additional strategies to improve global climate policy remain necessary to curb global emissions within this decade. Identifying and expanding good practice policy approaches, that have a substantial effect on emissions, would close part of the gap between current emission projections and pathways compatible with limiting temperature increase to 1.5 °C^16,17^.

Climate change mitigation efforts rely on a combination of policies, each of which have different effects^18^. Balancing these effects increases the chance that collective objectives are met and that instruments lead to net-positive outcomes^19^. Although an increase in the total number of policies help reducing emissions, the distribution of policy instruments across sectors probably influences their collective effect. We argue that empirical research focusing on these distributions enables identifying potential policy entry points to slow down projected emission growth.

In our research, we compared the number of policies in force across country groups to identify means to expand and improve climate policy. First, we evaluated whether the prevalence of policies is associated with lower projected emission change rates between 2021 and 2030. This clarifies that expanding climate policy adoption is desirable to slow down future emissions. Second, we compared the number of policies across country groups to identify areas with substantially fewer policies. This supports identifying means to expand climate policy adoption in line with best-performing countries.

We use a comprehensive policy dataset to calculate the number of climate policies in force, or policy density. This dataset has been used in previous publications and is periodically updated to reflect recent policy adoption^20,21^. We associate this dataset with emission projections developed between 2015 and 2021. Emission projections are based on the ‘current policy scenario’ developed in the Climate Action Tracker (CAT) project^22^. We analyse 40 countries (Supplementary Table 1), which together account for 84% of global emissions in 2019^23,24^.

In our research, we used linear regressions to investigate whether the total number of climate policies, or policy density, explains the variance in projected emission-change rate distributions. We also used clustering analysis and statistical significance tests to evaluate how policy density varies across country groups. The linear regression analysis clarifies whether a larger policy portfolio is associated with lower projected emissions. The country group comparisons add nuance by considering that countries are in different stages of climate change mitigation efforts. In this case, we compare the number of policies across groups to identify which ones are less prevalent in country groups with higher projected emission growth. We identified policy expansion entry points by benchmarking adoption against the best-performing country group (see the “Methods” section).

## Results

### Country clusters

We used a clustering approach to explicitly consider country differences without relying on development-based categorisations, such as Annex-I in the UNFCCC context. We instead used country characteristics closely related to their future mitigation efforts. We applied this approach because of our focus on projected emission change rates. Although current political economy constraints will likely remain relevant, we argue that countries’ future emissions are highly dependent on countries’ interpretation of their fair-share contribution to minimising the impacts of climate change, as outlined in the Paris Agreement^25^. We included historical GDP and emission per capita because they are important indicators to distribute future mitigation responsibilities and are also commonly mentioned in countries’ own mitigation targets to justify their ambition^26^. We also used countries’ projected emission change rates to account for the already expected direction of change. Collectively, these indicators also account for many other important country characteristics. For example, emission per capita is a measure of the emission intensity of the country. Therefore, it is affected by high dependency on fossil fuels for energy or economic development.

**Fig. 1:**
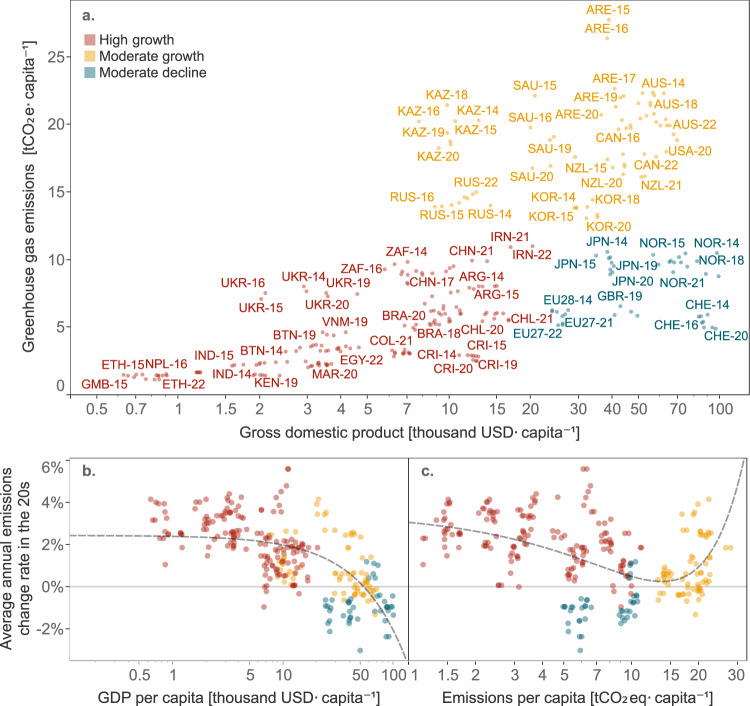
Clustering analysis results. Mind the log scale in all charts. The three letters represent the country’s ISO-3 code and the two digits the year of the data (historical GDP/capita, historical emissions/capita and projected average change rate developed in the respective year).

Our analysis identified three country clusters with distinct characteristics. The clusters are defined by countries’ historical emissions per capita, GDP per capita and projected emission change rates. We do not observe any overlap between groups across historical emissions and GDP per capita nor do we observe a change in the cluster the country belongs to over time (Fig. 1a). This indicates that the clusters are sufficiently heterogeneous and that the categorisation is time-invariant within the timeframe considered. GDP per capita and emission change rates show an inverse and monotonic relationship (*R*^2^ = 0.43; *p*-value < 0.01). Countries with higher economic capability tend to have lower emission change rates between 2021 and 2030 (Fig. 1b). The relationship between historical emission per capita and projected emission change rate is less significant (*R*^2^ = 0.25; *p*-value < 0.01) but is probably concave. Emissions in countries with both low and high per capita emission levels are projected to grow faster (Fig. 1c). The different clusters are described in more detail below.

The ‘high growth’ cluster includes 25 countries with a projected mean annual emission change rate of 2.3%—the highest among the three clusters. Its countries tend to have lower emissions and GDP per capita. It includes all low-income and some emerging economies, such as Argentina, Brazil, China, India, Indonesia, Mexico, South Africa and Turkey. Together the countries in this cluster represent approximately half of global emissions^23,24^. In the past two years, South Africa’s emission change rate is substantially lower than the other countries in the same cluster.

The ‘moderate growth’ cluster includes nine countries with a projected mean annual change rate of 0.9%. It is strongly defined by high historical emission per capita, which is for all countries above 13 tCO_2_eq in the period analysed. Together these countries represent almost a quarter of global emissions. This cluster includes several developed countries, including some that have curbed emissions growth, such as Australia, Canada, South Korea and the United States. It also includes a few countries with increasing emissions, such as Saudi Arabia and Kazakhstan. Despite these differences, fossil fuel dependency for economic or energy purposes plays a substantial role in this cluster, which includes several of the world’s top fossil fuel producers^27^.

The ‘moderate decline’ cluster includes six of the countries analysed and has a projected mean annual emission change rate of −0.9%. This cluster is characterised by medium-to-high GDP and medium emissions per capita. Together the countries in this cluster represent approximately one-tenth of global emissions. Japan, Switzerland, Norway, the European Union as a group and the United Kingdom, after its exit from the European Union, all show declining emissions in the period analysed. Singapore is the only country in this cluster where emissions still increase.

### Policy density is associated with projected emissions

**Fig. 2:**
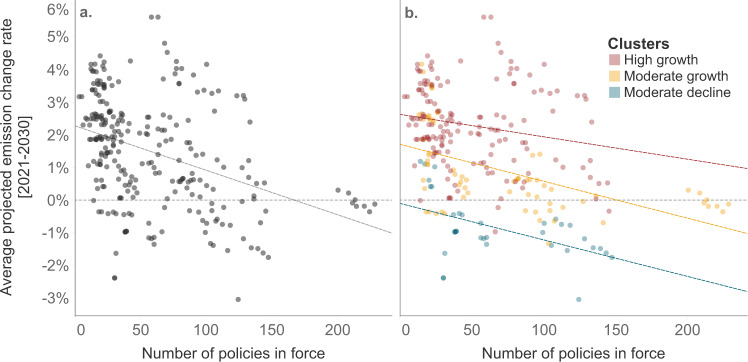
Relationship between average annual projected emission change rates and a number of policies. Countries with more climate policies have lower projected emission change rates (**a**). This result is valid across clusters although to a different degree (**b**). Results are statistically significant despite variance.

We find that countries with more policies have lower projected emissions up to 2030. The total policy density is associated with a lower average emission change rate between 2021 and 2030 (Fig. 2a). These results are robust when controlling for the rule of law, per capita values for historical emissions and GDP, and the number of high-impact policies. Also, although the magnitude of the effect varies across clusters, policy density is associated with lower emissions growth across all clusters (Fig. 2b).

In our regression analysis, we controlled for the number of high-impact policies and the rule of law, even though we did not explicitly account for the stringency of policies in force (Supplementary Table 2). The number of high-impact policies controls the prevalence of policies that are considered to have a substantial effect on emissions or are prominent in the national policy debate. These policies are often also used in the quantification of future emissions. Rule of law is used as a proxy for the quality of law-making and enforcement. Including these variables accounts for potential bias in the development of current policy projections and varying degrees of potential policy implementation. Both these variables are associated with projected emissions. Stronger rule of law is associated with lower projected emissions. However, the number of high-impact policies is not. The number of high-impact policies is substantially lower than the total number of policies in force and our findings suggest that a higher number of these policies alone is insufficient to reduce projected emissions. We also control for GDP and emissions per capita, since these two variables are important to contextualise future mitigation efforts. We find that the policy density is important to reduce future emissions in countries independently of their emissions intensity per capita and economic capability. However, countries with higher economic capability tend to have lower projected emissions.

No other study analysed the effect of policies on emission projections using similar methods, but some have analysed the effect of policies on historical emissions. Eskander and Fankhauser found that countries’ climate policy portfolio reduced annual emissions change rate by 0.8 p.p. between 1990 and 2016^7^. Our average-sized portfolio is expected to reduce annual emissions growth by 0.8 p.p. by 2030. We note that substantial differences in the size of the average portfolio exist between the two studies due to differences in climate policy definition^21^. Also, closed-form functions describing the effect of policies on emissions are only relevant for comparison purposes as actual causal relationships are substantially more complex. Nonetheless, both studies suggest a statistically significant effect in the same direction and of similar magnitude.

Although policies reduce projected emission growth, adoption varies within countries. Policy density is not homogeneous across sectors (Supplementary Fig. 1). The agriculture sector has the fewest adopted policies (mean: 4). This probably contributes to the lack of progress in reducing agricultural emissions^28^. Countries adopt most policies (mean: 21) in the electricity and heat supply sector. This is partly explained by the earlier start of climate policy adoption in this sector^29^. We also find a strong relationship between the number of policies in the electricity and heat supply and other sectors (Supplementary Fig. 2). In other words, countries with many policies in this sector tend to have many policies in others too. This supports the existence of positive cross-sectoral policy feedback lowering adoption barriers across sectors. These findings combined suggest that the electricity and heat sector has been an entry point for policy expansion.

Similarly, the number of policies across instrument types varies (Supplementary Fig. 1). Market-based (mean: 2) and voluntary approaches (mean: 3) are the least adopted instrument types. Their low prevalence is partly explained by policy instrument sequencing since both instruments are often implemented last across countries^30^. These two instrument types are therefore more common in mature climate policy portfolios. Other instrument types are substantially more prevalent. For example, countries adopt more fiscal and financial incentives (mean: 15) and regulatory instruments, such as codes and standards (mean: 12). These two instrument types rely on very distinct mechanisms. While the former intends to provide benefits to low-carbon interventions, the latter aims to penalise polluters. Literature investigating instrument sequencing suggests that benefits are introduced first and then are followed by regulatory policies^31^. Now both approaches are almost equally prevalent.

Considering the relationship across policy instrument types, we observe that countries with many Research & Development (R&D) and information policies tend to adopt more policies across instrument types (Supplementary Fig. 2). R&D policies are insufficient to reduce emissions alone but foster innovation, which helps reducing mitigation options’ costs^32^. Information and education policies support behavioural changes, the adoption of low-carbon technologies and lower adoption barriers for more stringent climate policies^11^. These relationships suggest that such policies represent important means to expand climate policy adoption.

Policy density across mitigation areas also indicates that some are more prevalent than others. Countries adopt more policies related to energy efficiency (mean: 32) and renewable energy (mean: 26) in comparison to the other three mitigation areas analysed. Other studies also found that countries with declining historical emissions have a substantially higher number of renewables and energy efficiency policies^33^.

### Entry points to expand climate policy adoption

**Fig. 3:**
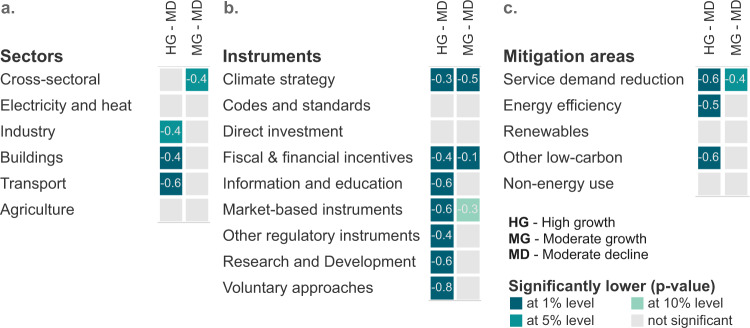
Comparing policy density to moderate decline cluster. Results from Mann–Whitney *U* one-tailed test comparing policy density across sectors (**a**), policy instrument types (**b**) and mitigation areas (**c**). Colours indicate whether policy density is significantly lower when compared to the ‘moderate decline’ cluster. Labels present the percentage difference of the medians. For example, the median number of climate strategies in the ‘high growth’ and ‘moderate growth’ clusters are respectively, 30% and 50% lower compared to the ‘moderate decline’ cluster.

Considering that an increase in the number of policies is associated with lower projected emissions growth, we aim to identify potential entry points to expand policy adoption using countries with a moderate decline in emissions as a reference. We compared policy density in the ‘high growth’ and ‘moderate growth’ clusters to that of the ‘moderate decline’ cluster (Fig. 3). Significance statements in the text are based on *p*-value results from the non-parametric comparison of policy density distributions.

The ‘high growth’ cluster has the highest average emission change rate expected in the coming decade and has significantly fewer policies across almost all sectors, policy instrument types and mitigation areas (Fig. 3).

Countries in the ‘high growth’ cluster adopt fewer energy-demand-related policies (dark blue squares in Fig. 3a). Energy-related sectors are responsible for a large share of ‘high growth’ countries’ historical emissions^28^. However, the average number of policies in transport, buildings and industry is in each sector approximately half compared to the ‘moderate decline’ cluster. Although policy density is lower in some sectors, countries in this group adopt similar numbers of electricity and heat supply, agriculture, and cross-sectoral policies. This suggests that expanding policy adoption in these sectors is not as critical as in energy demand, although it would probably still lead to positive outcomes. The disproportionally lower number of policies targeting energy demand sectors represents a clear entry point to further reduce emissions in these countries.

Countries in the ‘high growth’ cluster also have a significantly lower number of policies in almost all policy instruments (Fig. 3b). This indicates that countries in this cluster can still substantially improve the size and diversity of their climate policy portfolio.

The ‘high growth’ cluster adopts less than half of the number of information and education, R&D, market-based and voluntary instruments. Some of these differences are anticipated. Voluntary approaches and market-based instruments are often implemented latest in policy instrument sequencing^30^. Annex-I countries, which have older commitments to reduce emissions in comparison to countries in the ‘high growth’ cluster, started implementing climate policies earlier. This contributes to a more diverse set of policies in force. Research and development policies are also expected to be more prevalent in these countries, which spend more on research^34^. However, we also observe significantly fewer information and education policies, which constitute a low-hanging fruit approach to expand policy adoption in this cluster.

Countries in the ‘high growth’ cluster also have significantly fewer policies across all mitigation areas, except non-energy and renewables (Fig. 3c). The number of policies is substantially and significantly lower in energy efficiency, service and demand reduction policies and low-carbon alternatives to increase renewable energy supply. Demand-side policies, including energy efficiency, reduce greenhouse emissions and has numerous benefits to other development goals^35^. Their adoption represents a clear strategy to improve climate change mitigation efforts. Other low-carbon alternatives, such as nuclear energy, and fuel switches, such as coal phase-out policies, are also important and significantly lower in the ‘high growth’ cluster^36,37^.

Countries in the moderate growth cluster have succeeded in slowing down projected emissions, which are still not declining despite the relatively high capability (GDP per capita) and high responsibility (emissions per capita). This cluster has, in general, more policies in force compared to the ‘high growth’ cluster but a significantly lower number of policies in some policy instrument types and mitigation areas when compared to the ‘moderate decline’ cluster.

Countries in the ‘moderate growth’ cluster adopt a similar number of sectoral policies when compared to ‘moderate decline’ but have a less integrated approach to mitigation efforts, due to fewer cross-sectoral and framework policies. Cross-sectoral policies, per definition, are those that target multiple sectors or provide a framework policy for climate change mitigation. Countries in this cluster have on average 40% fewer cross-sectoral policies adopted compared to ‘moderate decline’ countries. This result is supported by the significantly fewer climate strategies, which frame mitigation commitments and efforts and indicate the scale of effort necessary^38,39^.

The ‘Moderate growth’ cluster also has a significantly lower number of fiscal and financial incentives and market-based instruments compared to the ‘moderate decline’ cluster, albeit the latter at a lower significance level. However, they have more regulatory instruments, direct investments, information and education programmes, voluntary approaches, and R&D policies. The comparison of policy instruments across these two clusters does not yield substantial insights, except for indicating that countries adopt policies using different approaches and that improving the number of market-based instruments and fiscal and financial incentives will probably improve mitigation efforts. Like the ‘high growth’ cluster, the ‘moderate growth’ cluster has a significantly lower number of policies in low-carbon and service demand reduction areas.

## Discussion

Our research contributes to the comparative climate-policy literature. Here, we use statistical methods to associate policy adoption with emission projections up to 2030. We find that increasing the number of policies in force is associated with slower emissions growth or faster emissions decline. By comparing distributions of policies, we account for national differences and obtain more statistically robust results than when comparing individual countries. The empirical evidence presented here indicates common approaches associated with emissions reductions and potential entry points to expand climate policy. Overall, considering the distribution of policies across sectors, policy instrument types and mitigation areas improves our understanding of the effect of climate policies.

We find that expanding climate policy adoption is associated with stronger climate change mitigation. Countries with more policies in force have lower projected emission change rates, independently of their historical emissions and GDP per capita and of the number of high-impact policies. However, GDP per capita is still strongly associated with emission change rates: high-income countries are more likely to decline emissions. The addition of policies slows down emissions whether a country is starting their mitigation efforts or already shows declining emissions. This evidence suggests that larger and more comprehensive climate policy portfolios are conducive to emissions reductions.

We do not suggest that countries should adopt more policies independently of their content but find that increasing the number of policies increases the probability that these policies collectively reduce emissions. Different causal mechanisms are probably at play. A few stringent policies might be responsible for a large share of the observed effect^3^ while weaker policies play a supportive role, e.g. by enabling their adoption. Alternatively, weaker policies might collectively cause the observed effect by reinforcing each other and balancing policymaker’s priorities^40^. The dominant mechanism remains unclear. More research on the interaction between climate policies, instead of their individual effect, is needed to explore the mechanisms leading to impact.

In our research, we identified potential policy entry points for policy expansion. Policies that aim to reduce demand beyond energy efficiency improvements (e.g., policies to reduce energy needs via improved urban planning) and to set climate change mitigation strategies are significantly and substantially fewer in countries that fail to decline projected emissions. We also find that countries with many policies targeting electricity and heat supply tend to have multiple policies in other sectors too. This, combined with the evidence that countries’ started climate policy adoption in this sector, suggests that addressing electricity and heat supply is a good entry point for sectoral policy adoption, especially in countries with fewer policies in force. These options represent clear opportunities to expand and improve climate policy adoption across countries.

However, countries at different stages of mitigation would benefit from differentiated approaches to expand their policy portfolios. Countries with fast-growing emissions have a lower number of policies in almost all sectors, policy instrument types and mitigation areas. While some of these are expected, key options to expand climate policy adoption include: education and information policies and programmes and policies that address energy use sectors. Countries that stalled their emissions growth have a similar number of policies compared to countries with declining emissions. A key difference is their significantly lower number of cross-sectoral, framework policies, such as climate strategies.

Our findings do not mean that expanding specific policy types alone will reduce emissions. All results must be analysed in the context of the policy portfolio. Research indicates that a progression of policy adoption over time exists^41^. Market-based instruments are usually the last type to be adopted in major emitting economies^30^. Cross-sectoral policies are also adopted later than sector-specific ones^29^. Finding that cross-sectoral and market-based instruments are lacking in countries with increasing emissions implies that these policies alone reduce emissions or that portfolios that contain these policies reduce emissions. Considering that the former mechanism is valid supports the expansion of these specific policy instruments. Considering that the latter mechanism is valid builds evidence of which characteristics of a mature climate policy portfolio contribute to slower projected emission change rates. Both interpretations generate valuable insights in the context of policy expansion, which remains fundamental to address climate change.

Climate change is a highly complex problem that contains multiple interdependencies with other societal issues^42^. Stakeholders have multiple, often value-laden perspectives about the problem’s relevance and the choice of solutions^43^. This implies the existence of diverse policy objectives intended to address conflicting viewpoints, foster agreement and enable coherent action^44^. Therefore, the adoption of multiple policy instruments is key to addressing multiple objectives^45,46^. Additionally, adopting multiple instruments aid in managing future uncertainty, addressing diverse market failures and improving governance^46^. Different studies find that larger policy portfolios contribute to sustainable transitions^47,48^. Here, we provided empirical evidence that larger and more comprehensive climate-policy portfolios reduce projected emissions growth.

However, expanding the number of climate policies has diverse policymaking implications. For example, expanding policy adoption leads to additional feedback effects^49^. Attention to this feedback during the policy formulation process is fundamental to ensure positive outcomes^50^. Parallel development of the administrative capacity to implement policies is also a condition to harness the positive effects of policy expansion^51^. However, we argue that policymakers are not required to anticipate all potential effects to implement climate policies, since sub-optimal policies combined can also lead to substantive emissions decline^52^. Also, policy portfolios result from a process of policy and political change^53^. Their development implies some degree of patching existing portfolios through policy expansion and dismantling^54,55^. Although re-structuring policy portfolios poses its own challenges, progressively improving solutions helps to avoid the risks of failing in the pursuit of single, first-best solutions^56^. Further research on the policymaking process, considering policy expansion implications, will probably help accelerate climate policy adoption.

Our research is subject to distinct limitations, some of which we discuss here. For example, it implicitly compares policy adoption to countries with the fastest emissions decline. However, their average decline rate is insufficient to meet the collective goals of the Paris Agreement^57^. This indicates that emissions reductions will remain insufficient even if countries replicate these policy approaches. Implementing some of the findings in this research will result in incremental improvements at best and must be combined with other interventions to keep the goals of the Paris Agreement within reach.

Additionally, investigating the effect of climate policies on emission projections developed using adopted climate policies potentially may lead to bias in the estimates. In other words, when policies are used to develop emission projections, they are by design associated with those projections. To this, we respond using two main arguments. First, we investigated the effect of the size of the policy portfolio, or policy density, on emission change rates. The policies used as input in these quantification efforts may or may not have a relationship with policy density. This effect is potentially concerning if all policies in the portfolio are used as input for the emissions modelling, which is not the case. Emission projections under current policies are based on the quantification of a subset of high-impact policies. Here, we also included a proxy for these quantifiable policies as a control variable and found that the total number of policies remains statistically associated with projected emissions. Second, the effects observed on emission projections are also observed on historical emissions. This shows that even historical emissions, which are not resulting from modelling exercises, are similarly associated with policies adopted. The use of projections enables analyses of the longer-term effect of climate policies and is, therefore, more adequate to analyse policy adoption since the Paris Agreement. We find that the relationship between the total policy density and future emission growth rates remains valid, even when projections are based on adopted policies.

Despite the increase in the number of policies adopted in the previous decades, countries must adopt new climate policies. Even countries with a higher number of policies fail to decrease emissions at the necessary rate to meet the collective goals of the Paris Agreement. Improving mitigation efforts is thus essential considering the urgency to reduce global emissions. Expanding climate policy adoption to align with historical best-in-class approaches presents a clear strategy to improve mitigation efforts. Although policy adoption differences exist between countries, many opportunities to expand policy adoption remain. Worldwide emissions can well be reduced with already existing mitigation options.

## Methods

Our analysis relies on panel data for 40 countries between 2014 and 2022. We included one data point for each country and year when emissions were projected, whenever data were available. The use of panel data improves our statistical findings. The sample used in this research consists of 263 data points.

### Emissions data

We used emission projections developed under the CAT project, which provides yearly updates to its ‘current policy scenario’ for the countries analysed^22^. CAT data were used in many scientific publications (e.g., refs. ^58,59^) and is a central input to the UNEP Emissions Gap Report^60^. These projections are based on the full implementation of selected climate policies in force at the time the projections were developed. They constitute our best-available consistent estimate of policies’ effect on future emissions considering present information for multiple countries. Emission projections exclude emissions from land use, land-use change and forestry (LULUCF).

CAT projections depart from country-specific reference scenarios, that are adjusted to include the effect of individual policies^3^. For the reference scenario, CAT relies on most recent government documents, such as a Biennial Update Reports (BUR) submitted to the UNFCCC, or on analyses from authoritative organisations, such as the International Energy Agency^61,62^. The choice of reference scenario depends on many factors such as the coverage of policies and assumptions regarding key emission drivers. When the reference scenario includes only energy-related CO_2_ projections, projections are complemented to ensure coverage of all emissions sources, including, for example, the US Environmental Protection Agency (EPA) report for non-CO_2_ emissions^63^.

When a recently adopted relevant policy is outside the scope of the reference scenario, its effect is included with add-on calculations. For example, CAT relies on the business-as-usual scenario from the Asia Pacific Energy Research Centre to estimate emission projections for Indonesia^64^. The scenario estimates that coal will represent 51% of national electricity generation by 2030. However, Indonesia’s official ten-year electricity supply plan indicates that coal will still represent 64% of electricity generation by the same year^65^. To account for this policy, CAT estimated emissions associated with electricity generation considering the total electricity demand presented in the official plan and applying emission factors for the relevant fossil technologies. They complement electricity-only estimates by assuming other sectors follow the growth projected in Indonesia’s latest BUR. The different scenarios are included in the projections analysed here as a range of emissions up to 2030. Similar approaches are taken for all countries analysed across the years.

We calculated the annual emission change per country between 2021 and 2030 and then averaged the results. The focus on average emission change rates highlights the expected dynamic in the coming decade instead of absolute changes in emission levels. In this calculation, we removed outliers (5−95 percentiles), such as abrupt change resulting from economic lockdown measures in response to the COVID-19 pandemic. These outliers were removed for each country’s projected annual change rate distribution before calculating the mean decadal change rate.

### Policy data

Policy data were extracted from the Climate Policy Database^20^. As of the end of 2022, this database included over 3000 national climate policies for the countries analysed. The policy database contains policies that affect country’s long-term emissions, even if policies do not have an explicit climate change mitigation objective. Our analysis includes national policies only. Considering the sectoral scope of emissions projections, we excluded LULUCF-related policies.

The database includes categorisation for each policy in terms of policy instrument types, sectors and mitigation areas. Policy instruments, such as voluntary approaches or subsidies, are tools used to implement policies and constitute a link between policy objectives and implementation^66^. The database uses a list of policy instrument types from the International Energy Agency^67^ that was adapted to better reflect climate-policy-related instruments. It also includes, for example, climate strategies, which are economy-wide framework policies that support the coordination of mitigation efforts across sectors. Mitigation areas are broadly defined as distinct strategies to reduce emissions, such as supporting renewable energy or energy efficiency. The taxonomy used in this research is introduced in Nascimento et al.^29^. but summarised in the Supplementary Methods. The database also includes a field that identifies high-impact policies. The high-impact categorisation reflects country experts’ expectations about policies effect and indicates policies that were often used to create the current policy projections.

In this research, the term policies refer to laws, legislations, executive orders, or their equivalent. Each policy is one entry in the database but can be coded as multiple policy instrument types, sectors and mitigation areas depending on its scope. For example, Argentine Law No. 27,640 on biofuels establishes a biofuel blending mandate on gasoline and diesel^68^. This policy is coded in multiple sectors since the mandate affects energy end-use sectors, such as transport and industry. Similarly, the Brazilian Law No. 13,755 aims to improve the energy efficiency of vehicles sold within the country^69^. It establishes mandatory requirements to manufacture or import vehicles in the country and offers tax reliefs for companies that prove research and development spending in line with the goals of the law. This law is coded as both a fiscal incentive and as a standard.

In our research, we focus on policy density, which describes the level of policy activity within a country^21^. Here, policy density was measured as the number of policies in force, as it is usually implemented in the relevant literature^54^. We do not explicitly consider the stringency or intensity of policies in force. This concept relates to the implementation of policies. It is associated with the resources mobilised, both from institutional and policy design perspectives, to implement policy instruments^70^. However, its operationalisation is not straightforward. Some researchers create metrics or indexes to assess the stringency of the policy portfolio to rank or compare countries^71,72^. In other cases, the stringency of individual policies is determined by their own target indicators^73^. This latter operationalisation of stringency depends on clear impact indicators and national counterfactual scenarios, which are often unavailable^74^. These different evaluation approaches also affect the stringency of distinct policy instrument types. For example, carbon pricing instruments are probably more cost-effective than regulatory instruments^11^. Therefore, when evaluating carbon pricing’s stringency based on this criterion, they perform better. However, a review of ex-post analyses suggests that they have a limited effect on reducing emissions^75^. Conclusions derived from different approaches to assess policy stringency are not necessarily robust across countries and a common metric to measure stringency is unavailable^76^.

We, therefore, test whether policy density alone explains projected emissions, without addressing the difficult issue of stringency. Additionally, although the stringency of policies matters, climate policies are missing in many important areas^29^. Expanding countries’ policy portfolios ensures that existing options to mitigate climate change are in place. Increasing policies’ stringency and expanding climate policy are both necessary to improve mitigation efforts. Our research helps to identify areas that are disproportionally unaddressed and potential policy expanstion entry points to improve national and global climate policy.

To characterise policy adoption, we calculated the total policy density as the count of distinct policies. This includes the stock of climate policies in force at the respective date, not only policies adopted within the period analysed. Since policies are not homogeneously distributed in a country, we also calculated policy density across sectors, mitigation areas and policy instrument types. Instead of counting the total number of policies, we calculated, for example, the number of policies in each sector, which corresponds to the policy density of each sector.

### Other data

National population data are based on the United Nations World Population Prospects and especially its medium fertility scenario^77^. We calculated historical levels of emissions per capita based on CAT emissions and UN population estimates. We used historical GDP per capita from World Bank^78^. The rule of law used as a control variable in the regression analysis is taken from the Worldwide Governance Indicators database^79^.

### Cluster analysis

We clustered countries based on their projected emission change rate, historical per capita gross domestic product (GDP) and historical per capita emission levels. This initial step is used to identify groups in the data based on country characteristics. We used the *k*-means clustering algorithm, that aims to identify groups in data by minimising within-group variance^80^ and chose the number of clusters that maximised the Calinski–Harabasz score^81^.

### Linear regression

We modelled the relationship between policy density and projected emission change rates using linear regressions. We used the total policy density as the independent variable and the projected mean annual project emission change rate as the dependent variable. We modelled this relationship using Ordinary Linear Regressions with robust errors. This choice is based on the result of Breusch–Pagan tests that showed the presence of heteroskedasticity^82^. Before building the regression model, we harmonised the data using the *Z*-score for each data point. This score measures the number of standard deviations by which the value differs from the distribution mean.

We controlled for historical GDP and emissions per capita, as described above. We also control for the number of high-impact policies, which are often used in the quantification of the emissions projections, to minimise hidden-variable bias and avoid endogeneity related to the development of current policy projections. Additionally, we controlled the rule of law. Although we did not explicitly account for the stringency of policies in force, this variable measures to which extend the population of a country has confidence in societal rules, for example considering contracts and courts^79^. Similarly to Eskander and Fankhauser^7^, we used this variable as a proxy to control for the implementation effectiveness of the policies in force.

### Statistical tests

We compared policy density across country clusters to analyse in which cases policy density in countries with increasing emissions is lower compared to countries with decreasing emissions.

We divided the data into distinct samples, one for each country cluster and used the Mann–Whitney *U* test to compare policy density distributions. Mann–Whitney *U* is a non-parametric test that measures whether the probability that the distribution underlying two samples is the same under its null hypothesis^83^. We ran one-tailed tests to compare the policy density between clusters.

### Reporting summary

Further information on research design is available in the Nature Research Reporting Summary linked to this article.

## Supplementary information

SupplementaryMaterial

Reporting Summary

## Data Availability

Emission data used is publicly available for non-commercial purposes at www.climateactiontracker.org. Policy data is also publicly available at www.climatepolicydatabase.org. Additional datasets generated during and/or analysed during the current study are available from the corresponding author on reasonable request.

## References

[1] IPCC. Summary for Policymakers. In *Climate Change 2021: The Physical Science Basis. Contribution of Working Group I to the Sixth Assessment Report of the Intergovernmental Panel on Climate Change* (eds Masson-Delmotte, V. et al.) 3–32 (Cambridge University Press, 2021).

[2] IPCC. Summary for policymakers. In *Global Warming of 1.5* *°C. An IPCC Special Report on the impacts of global warming of 1.5* *°C above pre-industrial levels and related global greenhouse gas emission pathways, in the context of strengthening the global response to the threat of climate change* (eds Masson-Delmotte, V. et al.) 3–24 (Cambridge University Press, 2018).

[3] Fekete, H. et al. A review of successful climate change mitigation policies in major emitting economies and the potential of global replication. *Renew. Sustain. Energy Rev.* **137**, 110602 (2021).

[4] Friedlingstein, P. et al. Global carbon budget 2021. *Earth Syst. Sci. Data Discuss* **2021**, 1–191 (2021).

[5] Liu, Z., Deng, Z., Davis, S. J., Giron, C. & Ciais, P. Monitoring global carbon emissions in 2021. *Nat. Rev. Earth Environ.* **3**, 217–219 (2022). 35340723 10.1038/s43017-022-00285-w PMC8935618

[6] Peters, G. et al. Carbon dioxide emissions continue to grow amidst slowly emerging climate policies. *Nat. Clim. Chang.* **10**, 3–6 (2020).

[7] Eskander, S. M. S. U. & Fankhauser, S. Reduction in greenhouse gas emissions from national climate legislation. *Nat. Clim. Chang.* **10**, 750–756 (2020).

[8] Best, R., Burke, P. J. & Jotzo, F. Carbon pricing efficacy: cross-country evidence. *Environ. Resour. Econ.* **77**, 69–94 (2020).

[9] Best, R. & Zhang, Q. Y. What explains carbon-pricing variation between countries? *Energy Policy* **143**, 111541 (2020).

[10] Skovgaard, J., Ferrari, S. S. & Knaggård, Å. Mapping and clustering the adoption of carbon pricing policies: what polities price carbon and why? *Clim. Policy* **19**, 1173–1185 (2019).

[11] Dubash, N. K. et al. 2022: National and sub-national policies and institutions. In *IPCC, 2022: Climate Change 2022: Mitigation of Climate Change. Contribution of Working Group III to the Sixth Assessment Report of the Intergovernmental Panel on Climate Change* (eds Shukla, P. R. et al.) (Cambridge University Press, 2022).

[12] Dubash, N. K. Climate laws help reduce emissions. *Nat. Clim. Chang.* **10**, 709–710 (2020).

[13] IPCC. Summary for Policymakers. in *Climate Change 2022: Mitigation of Climate Change. Contribution of Working Group III to the Sixth Assessment Report of the Intergovernmental Panel on Climate Change* (eds. Shukla, P. R. et al.) (Cambridge University Press, 2022).

[14] Nascimento, L., Kuramochi, T. & Höhne, N. The G20 emission projections to 2030 improved since the Paris Agreement, but only slightly. *Mitig. Adapt. Strateg. Glob. Change* **27**, 39 (2022). 10.1007/s11027-022-10018-5 PMC9281192 35855774

[15] den Elzen, M. G. J. et al. Updated nationally determined contributions collectively raise ambition levels but need strengthening further to keep Paris goals within reach. *Mitig. Adapt. Strateg. Glob. Change* **27**, 33 (2022). 10.1007/s11027-022-10008-7 PMC9209833 35755269

[16] Baptista, L. B. et al. Good practice policies to bridge the emissions gap in key countries. *Glob. Environ. Change* **73**, 102472 (2022).

[17] van Soest, H. L. et al. Global roll-out of comprehensive policy measures may aid in bridging emissions gap. *Nat. Commun.* **12**, 6419 (2021). 34741020 10.1038/s41467-021-26595-z PMC8571395

[18] Peñasco, C., Anadón, L. D. & Verdolini, E. Systematic review of the outcomes and trade-offs of ten types of decarbonization policy instruments. *Nat. Clim. Chang.* **11**, 257–265 (2021).

[19] van den Bergh, J. et al. Designing an effective climate-policy mix: accounting for instrument synergy. *Clim. Policy* **21**, 1–20 (2021).

[20] NewClimate Institute. *Climate Policy Database* (NewClimate Institute, 2021); https://climatepolicydatabase.org/.

[21] Schaub, S., Tosun, J., Jordan, A. & Enguer, J. Climate policy ambition: exploring a policy density perspective. *Political Governance;* 10(6) *Explor. Clim. Policy Ambition* (2022).

[22] Climate Action Tracker. *Glasgow’s 2030 Credibility Gap: Net Zero’s Lip Service to Climate Action. Warming Projections Global Update* https://climateactiontracker.org/publications/glasgows-2030-credibility-gap-net-zeros-lip-service-to-climate-action/ (2021).

[23] FAO. *FAOSTAT Emissions Database* http://www.fao.org/faostat/en/#data (2023).

[24] Olivier, J. G. J. & Peters, J. A. H. W. Trends in global emissions of CO2 and total greenhouse gases: 2021. (PBL Netherlands Environmental Assessment Agency, 2021).

[25] UNFCCC. *Paris Agreement—Decision 1/CP*.*21—Report of the Conference of the Parties on its Twenty-first session*, *held in Paris from 30 November to 13 December 2015 Addendum Part two: Action taken by the Conference of the Parties at its Twenty-first session* http://unfccc.int/resource/docs/2015/cop21/eng/10a01.pdf (UNFCCC, 2015).

[26] Rajamani, L. et al. National ‘fair shares’ in reducing greenhouse gas emissions within the principled framework of international environmental law. *Clim. Policy* **21**, 983–1004 (2021).

[27] BP. *Statistical Review of World Energy* https://www.bp.com/content/dam/bp/business-sites/en/global/corporate/pdfs/energy-economics/statistical-review/bp-stats-review-2021-full-report.pdf (2021).

[28] Lamb, W. F. et al. A review of trends and drivers of greenhouse gas emissions by sector from 1990 to 2018. *Environ. Res. Lett.* **16**, 073005 (2021).

[29] Nascimento, L. et al. Twenty years of climate policy: G20 coverage and gaps. *Clim. Policy* **22**, 158–174 (2022).

[30] Linsenmeier, M., Mohommad, A. & Schwerhoff, G. Policy sequencing towards carbon pricing among the world’s largest emitters. *Nat. Clim. Chang.* **12**, 1107–1110 (2022).

[31] Meckling, J., Sterner, T. & Wagner, G. Policy sequencing toward decarbonization. *Nat. Energy* **2**, 918–922 (2017).

[32] Bosetti, V., Carraro, C., Duval, R. & Tavoni, M. What should we expect from innovation? A model-based assessment of the environmental and mitigation cost implications of climate-related R&D. *Energy Econ.* **33**, 1313–1320 (2011).

[33] Le Quéré, C. et al. Drivers of declining CO2 emissions in 18 developed economies. *Nat. Clim. Change* **9**, 213–217 (2019).

[34] World Bank. Research and development expenditure (% of GDP) [Dataset]. *World Development Indicators* https://data.worldbank.org/indicator/GB.XPD.RSDV.GD.ZS (2022).

[35] Creutzig, F. et al. Demand-side solutions to climate change mitigation consistent with high levels of well-being. *Nat. Clim. Change* **12**, 36–46 (2022).

[36] Fell, H., Gilbert, A., Jenkins, J. D. & Mildenberger, M. Nuclear power and renewable energy are both associated with national decarbonization. *Nat. Energy* **7**, 25–29 (2022).

[37] Green, F. & Denniss, R. Cutting with both arms of the scissors: the economic and political case for restrictive supply-side climate policies. *Clim. Change* **150**, 73–87 (2018).

[38] Iacobuta, G., Dubash, N. K., Upadhyaya, P., Deribe, M. & Höhne, N. National climate change mitigation legislation, strategy and targets: a global update. *Clim. Policy* **18**, 1114–1132 (2018).

[39] Dubash, N. K., Hagemann, M., Höhne, N. & Upadhyaya, P. Developments in national climate change mitigation legislation and strategy. *Clim. Policy* **13**, 649–664 (2013).

[40] Karlsson, M., Alfredsson, E. & Westling, N. Climate policy co-benefits: a review. *Clim. Policy* **20**, 292–316 (2020).

[41] Pahle, M. et al. Sequencing to ratchet up climate policy stringency. *Nat. Clim. Change* **8**, 861–867 (2018).

[42] Sun, J. & Yang, K. The wicked problem of climate change: a new approach based on social mess and fragmentation. *Sustainability* **8**, 1312 (2016).

[43] Head, B. W. & Alford, J. Wicked problems: implications for public policy and management. *Adm. Soc.* **47**, 711–739 (2013).

[44] Head, B. W. Forty years of wicked problems literature: forging closer links to policy studies. *Policy Soc.* **38**, 180–197 (2019).

[45] Tinbergen, J. *On the Theory of Economic Policy* (North-Holland Publishing Company, Amsterdam, 1952).

[46] Bouma, J. A., Verbraak, M., Dietz, F. & Brouwer, R. Policy mix: mess or merit? *J. Environ. Econ. Policy* **8**, 32–47 (2019).

[47] Rosenow, J., Kern, F. & Rogge, K. The need for comprehensive and well targeted instrument mixes to stimulate energy transitions: the case of energy efficiency policy. *Energy Res. Soc. Sci.* **33**, 95–104 (2017).

[48] Campbell, S. & Coenen, L. *Transitioning Beyond Coal: Lessons from the Structural Renewal of Europe’s Old Industrial Regions* https://coaltransitions.files.wordpress.com/2017/11/australian-coal-transition-industrialization-final.pdf (2017).

[49] Pierson, P. When effect becomes cause: policy feedback and political change. *World Politics* **45**, 595–628 (1993).

[50] Leipprand, A., Flachsland, C. & Pahle, M. Starting low, reaching high? Sequencing in EU climate and energy policies. *Environ. Innov. Soc. Transit.* **37**, 140–155 (2020).

[51] Limberg, J., Steinebach, Y., Bayerlein, L. & Knill, C. The more the better? Rule growth and policy impact from a macro perspective. *Eur. J. Political Res.* **60**, 438–454 (2021).

[52] Bertram, C. et al. Complementing carbon prices with technology policies to keep climate targets within reach. *Nat. Clim. Change* **5**, 235–239 (2015).

[53] Howlett, M. & Rayner, J. Patching vs. packaging in policy formulation: assessing policy portfolio design. *Polit. Gov.* **1**, 11–25 (2013).

[54] Knill, C., Schulze, K. & Tosun, J. Regulatory policy outputs and impacts: exploring a complex relationship. *Regul. Gov.* **6**, 427–444 (2012).

[55] Kern, F., Kivimaa, P. & Martiskainen, M. Policy packaging or policy patching? The development of complex energy efficiency policy mixes. *Energy Res. Soc. Sci.* **23**, 11–25 (2017).

[56] Levin, K., Cashore, B., Bernstein, S. & Auld, G. Overcoming the tragedy of super wicked problems: constraining our future selves to ameliorate global climate change. *Policy Sci* **45**, 123–152 (2012).

[57] Höhne, N. et al. Emissions: world has four times the work or one-third of the time. *Nature* **579**, 25–28 (2020). 32132686 10.1038/d41586-020-00571-x

[58] Höhne, N. et al. Wave of net zero emission targets opens window to meeting the Paris Agreement. *Nat. Clim. Change* **11**, 820–822 (2021).

[59] Rogelj, J. et al. Paris Agreement climate proposals need a boost to keep warming well below 2 °C. *Nature* **534**, 631–639 (2016). 27357792 10.1038/nature18307

[60] UNEP. *Emissions Gap Report 2022* https://www.unep.org/resources/emissions-gap-report-2022 (UNEP, 2022).

[61] UNFCCC. *Biennial Update Report submissions from Non-Annex I Parties* https://unfccc.int/BURs (UNFCCC, 2019).

[62] IEA. *2022**World Energy Outlook- WEO2022* (IEA, 2022).

[63] U.S. EPA. *Global Anthropogenic Non-CO2 Greenhouse Gas Emissions: 2015–2050*. Report EPA-430-R-19-010, Vol. 2013 (U.S. EPA, 2019).

[64] APERC. *APEC Energy Demand and Supply Outlook**—*7th edn, Vol. II (Asia Pacific Energy Research Centre, The Institute of Energy Economics, Japan, 2019).

[65] Republic of Indonesia. *Rencana Usaha Penyediaan Tenaga Listrik (RUPTL) 2021–2030* https://web.pln.co.id/statics/uploads/2021/10/ruptl-2021-2030.pdf (2021).

[66] Rogge, K. S. & Reichardt, K. Policy mixes for sustainability transitions: an extended concept and framework for analysis. *Res. Policy* **45**, 1620–1635 (2016).

[67] IEA. *Policy Database—Data & Statistics* https://www.iea.org/policies (International Energy Agency, 2020).

[68] Argentina. *Law 27,640*—*Biofuel regulation.* (National Congress, 2021).

[69] Federative Republic of Brazil. *Law No. 13,755—Establishing Mandatory Requirements for the Commercialization of Vehicles.* (National Congress, 2018).

[70] Schaffrin, A., Sewerin, S. & Seubert, S. Toward a comparative measure of climate policy output. *Policy Stud. J.* **43**, 257–282 (2015).

[71] Botta, E. & Kozluk, T. *Measuring Environmental Policy Stringency in OECD Countries. A Composite Index Approach* http://www.oecd-ilibrary.org/docserver/download/5jxrjnc45gvg.pdf?expires=1441637037&id=id&accname=guest&checksum=EA1C00E6E8BC500DA6645821A89FA27710.1787/5jxrjnc45gvg-en (2014).

[72] Burck, J. et al. *Climate Change Performance Index. Results 2022* https://ccpi.org/download/climate-change-performance-index-2022-2/ (2021).

[73] Roelfsema, M. et al. Developing scenarios in the context of the Paris Agreement and application in the integrated assessment model IMAGE: a framework for bridging the policy-modelling divide. *Environ. Sci. Policy* **135**, 104–116 (2022).

[74] Somanathan, E. et al. National and sub-national policies and institutions. climate change 2014: mitigation of climate change. *Contribution of Working Group III to the Fifth Assessment Report of the Intergovernmental Panel on Climate Change* (Cambridge University Press, 2014).

[75] Green, J. F. Does carbon pricing reduce emissions? A review of ex-post analyses. *Environ. Res. Lett.* **16**, 043004 (2021).

[76] Galeotti, M., Salini, S. & Verdolini, E. Measuring environmental policy stringency: approaches, validity, and impact on environmental innovation and energy efficiency. *Energy Policy* **136**, 111052 (2020).

[77] UN. *World Population Prospects 2019* https://population.un.org/wpp/Download/Metadata/Documentation/ (2019).

[78] World Bank. *GDP (constant 2015 US$) [Dataset]*. *World Development Indicators* (World Bank, 2022).

[79] Kraay, A., Kaufmann, D. & Mastruzzi, M. *The Worldwide Governance Indicators: Methodology and Analytical Issues*. Policy Research Working Papers (The World Bank, 2010).

[80] Arthur, D. & Vassilvitskii, S. *K*-Means++: the advantages of careful seeding. In *Proceedings of the 18th Annual ACM-SIAM Symposium on Discrete Algorithms* 1027–1035 (Society for Industrial and Applied Mathematics, 2007).

[81] Caliński, T. & Harabasz, J. A dendrite method for cluster analysis. *Commun. Stat.* **3**, 1–27 (1974).

[82] Breusch, T. S. & Pagan, A. R. A simple test for heteroscedasticity and random coefficient variation. *Econometrica* **47**, 1287–1294 (1979).

[83] Mann, H. B. & Whitney, D. R. On a test of whether one of two random variables is stochastically larger than the other. *Ann. Math. Stat.* **18**, 50–60 (1947).

