# Supplementary material for: Expanding climate policy adoption improves national mitigation efforts
Source: NPJ Clim Action. 2023 Jun 9;2(1):12. doi: 10.1038/s44168-023-00043-8 (PMC10250858; doi:10.1038/s44168-023-00043-8)
Supplement: Supplementary file 1 — SupplementaryMaterial [file 44168_2023_43_MOESM1_ESM.pdf]

Supplementary Material: Expanding climate policy adoption improves national mitigation efforts

Supplementary figures

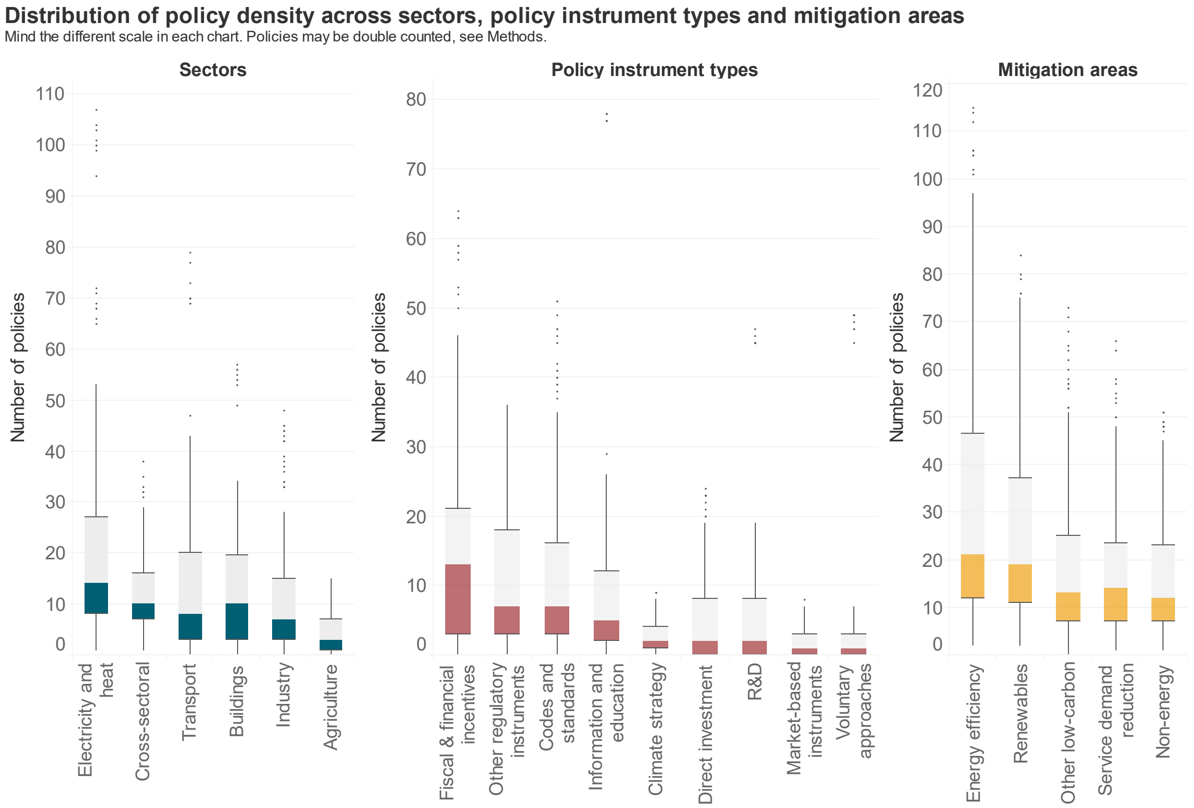

Supplementary Figure 1: Distribution of policies per sector, policy instrument and mitigation area.

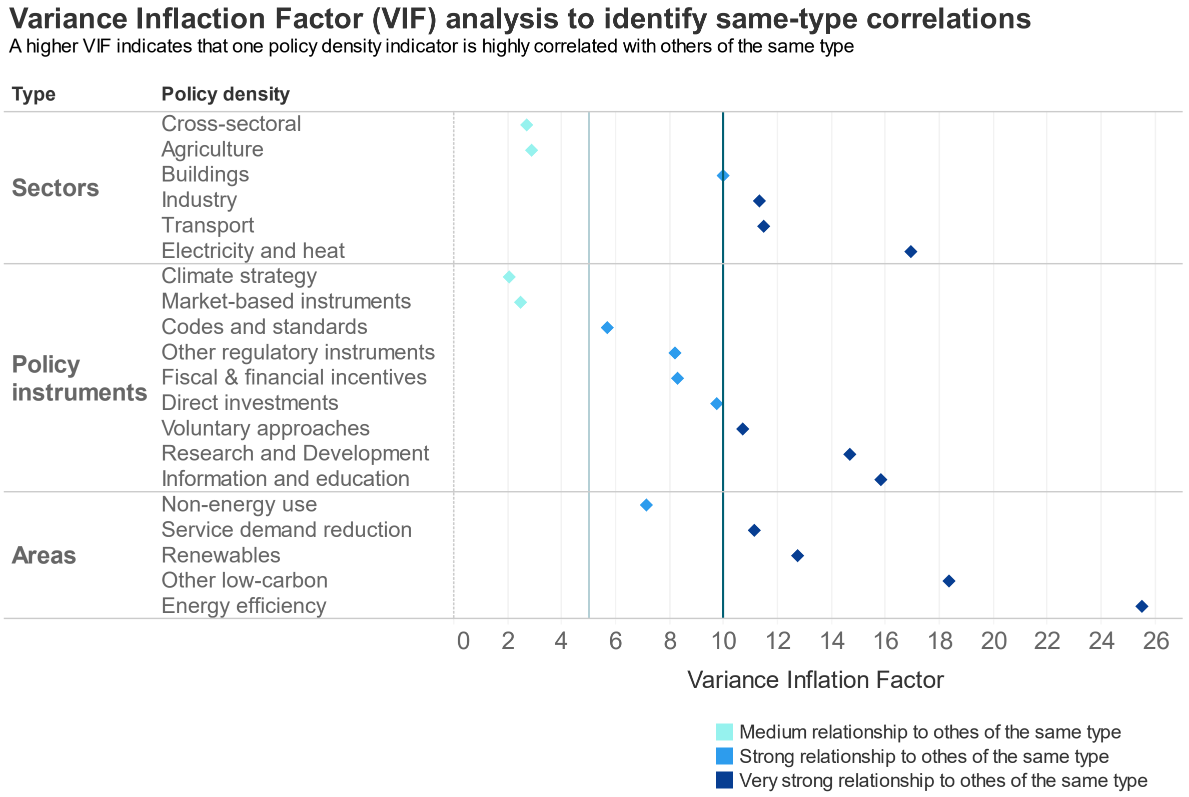

Supplementary Figure 2: Variance inflation factor (VIF) of distinct policy density indicators. The figure indicates that a high number of electricity and heat policies is associated with a high number of policies in other sectors. While a correlation matrix measures one-on-one relationship between policy density indicators, the VIF measure one-to-many relationships and summarises the information in one single value. Here, we use typically-used VIF cut-off values for reference[1].

Supplementary methods

This section presents a summary of the typology used in the research and example of policies included. This section is adapted from Nascimento et. al[2].

Policy instrument types

The original typology includes additional instruments since these categories could be further disaggregated. However, further disaggregation resulted in samples too small for statistical analyses.

Policy instrument type	Description
Climate strategies	Includes, for example, formal and legally binding climate strategies and climate change strategies which are not enshrined in law but are rather adopted through policy documents published by government agencies.
Codes and standards	Codes and standards are a very prominent sub-category of regulatory instruments. They refer to, for example, building codes and standards, industrial air pollution standards, product standards, vehicle fuel-economy and emissions standards

Policy instrument type	Description
Direct investments	Direct investments differ from fiscal and financial incentives because they refer to direct investments by national governments. For example, they include direct transfer of funds from national to sub-national governments for activities that have a potential to reduce the region’s GHG emissions. Also, infrastructure investments with a mitigation component would also be considered a direct investment.
Fiscal and financial incentives	Fiscal and financial incentives are a specific type of economic instruments that indirectly provide incentives for measures but do not establish a market-based mechanism. Some examples are feed-in tariffs, CO_2 and other taxes and subsidies.
Information and education	Refer to policies aimed are informing or educating users. Some examples include consumer-oriented labelling schemes that provide a rating of good or service against a pre-determined scale or that inform that good or products adapts to pre-defined minimum standards related to mitigation outcomes. It also includes schemes aimed at increasing access to information with potential positive mitigation outcomes, such as energy savings approaches or training programs for activities associated directly or indirectly with mitigation outcomes.
Market-based instruments	Includes multiple instruments such as: government-established emissions limits or caps on specific actors which can be traded to incentivize cost-effective emissions reductions; Scheme for the generation of tradable renewable energy certificates, or; schemes for generating tradable energy savings certificates produced by energy efficiency activities measured against a baseline.
Other regulatory approaches	Other regulatory approaches are those which do not fall into the codes and standards category, such as obligation schemes (e.g., mandatory requirement to comply with regular quotas for mitigation-related outcomes, such as yearly energy efficiency improvements for businesses).
Research and Development	Includes incentive schemes to accelerate the production of near-to-market technologies, nascent technologies with mitigation potential or the support for the implementation of pre-operational technologies or new uses of existing technologies.
Voluntary approaches	Voluntary approaches include partnerships between public and private actors for the implementation of mitigation-related activities or agreed voluntary commitments. It also includes several schemes to support voluntary activities by private actors, such as providing incentives to overcome split incentives (e.g., between landlord and tenant).

Sectors

Agriculture

Includes policies to increase sustainable practice in agriculture. Policies associated with sustainability standards for biomass used as a source for biofuels in other sectors are also included in this sector.

Buildings

Includes policies that target energy-use in buildings. These policies address building structure, appliances, cooking and heating/cooling devices. This sector also contains urban planning strategies.

Electricity and heat

Policies related to energy supply and enabling infrastructure, such as transmission and distribution grids, are included in this sector. However, policies related to fossil fuel exploration and production are included in the industry sector.

General

Cross-sectoral policies or policies that apply to any sector and that provide framing for or enable the implementation of other sectoral policies are included in this sector. These include, but are not limited to, national or sectoral climate strategies and Research and Development (R&D) policies.

Industry

Policies covering both energy-generation for own use and process-related emissions. This sector also includes policies related to other non-energy emissions. For example, emissions related to waste or fossil fuel exploration.

Transport

This sector includes policies related to all modes of land transportation and infrastructure programmes that might reduce transport needs (e.g., urban planning).

Mitigation areas

Energy service demand reduction and resource efficiency

Includes policies that indirectly reduce demand by supporting activity changes (e.g., reducing material use in manufacturing industries or developing urban planning strategies to minimize transport needs). These policies modify demand for goods and services by targeting choices/adoption of technology, consumption, behaviour, lifestyles, coupled production–consumption infrastructures and systems, service provision and associated socio-technical transitions" [3].

Energy efficiency

Includes policies to reduce national energy use. Energy efficiency policies include both framing policies with a goal to reduce energy consumption, such as energy efficiency targets, as well as policy options that support energy reductions in specific sectors, such as subsidies for energy efficient appliances or fuel consumption standards.

Renewables

Includes policies to support renewable energy technologies. This support might take a direct form, via subsidies or loans, or indirect, such as by developing grid infrastructure technology, that support the integration of high share of variable electricity generation technologies. This mitigation area includes policies that support the uptake of renewables in energy-use sectors, such as bio-energy mandates in in transport.

Other low-carbon technologies and fuel switch

Includes policies that support non-renewable, low-carbon technologies and options that impose limitations on the use of emissions-intensive technologies. Low-carbon technologies include support for nuclear electricity and fuel switch include for example coal and oil phase out policies. In this mitigation area we also include policies that support carbon dioxide removal technology development.

Non-energy

Includes policies that reduce non-energy related emissions. For example, policies to reduce emissions associated with fossil fuel exploration and production, industrial processes and the ban of fluorinated greenhouse gases.

Supplementary tables

Supplementary Table 1: ISO3 of the countries analysed.

ARE	CRI	KAZ	PHL
ARG	ETH	KEN	RUS
AUS	EU27/28 (not an ISO code)	KOR	SAU
BRA	GAB	MAR	SGP
BTN	GBR	MEX	THA
CAN	GMB	NGA	TUR
CHE	IDN	NOR	UKR
CHL	IND	NPL	USA
CHN	IRN	NZL	VNM
COL	JPN	PER	ZAF

Supplementary Table 2: Regression results. In all models we present the results including and excluding control variables. Considering the sample size, we focus on the total results but present the regression per cluster for transparency. The number of policies remain statistically associated with the projected emission change rates across clusters.

	Dependent variable: Mean projected emissions growth rate, 2021-2030							
	Total control	Total	Moderate decline control	Moderate decline	Moderate growth control	Moderate growth	High growth control	High growth
Number of Policies	-0.28***	-0.39***	-1.33***	-0.51***	-0.23*	-0.56***	-0.72***	-0.20**
	(0.08)	(0.05)	(0.14)	(0.15)	(0.13)	(0.09)	(0.14)	(0.08)
GDP per capita	-0.56***		-0.06		-0.36		-0.51***	
	(0.08)		(0.18)		(0.25)		(0.10)	
Emissions per capita	0.06		0.58***		0.53***		0.09	
	(0.05)		(0.19)		(0.11)		(0.10)	
Rule of law	-0.10		-0.82***		-0.14		0.02	
	(0.08)		(0.27)		(0.17)		(0.07)	
Number of high-impact policies	0.08		0.02		-0.04		0.64***	
	(0.07)		(0.18)		(0.11)		(0.14)	
Observations	263	263	35	35	72	72	156	156
R^2	0.51	0.15	0.76	0.26	0.56	0.31	0.31	0.04
Adjusted R^2	0.50	0.15	0.72	0.24	0.53	0.30	0.28	0.03
Residual Std. Error	0.71 (df=257)	0.93 (df=261)	0.54 (df=29)	0.88 (df=33)	0.69 (df=66)	0.84 (df=70)	0.85 (df=150)	0.99 (df=154)
F Statistic	72.94*** (df=5; 257)	68.17*** (df=1; 261)	43.26*** (df=5; 29)	11.23*** (df=1; 33)	16.65*** (df=5; 66)	39.12*** (df=1; 70)	24.18*** (df=5; 150)	6.34** (df=1; 154)

Note: *p<0.1; **p<0.05; ***p<0.01

Supplementary references

1. Craney, T. A. & Surles, J. G. Model-Dependent Variance Inflation Factor Cutoff Values. *Qual. Eng.* **14**, 391–403 (2002).
2. Nascimento, L. *et al.* Twenty years of climate policy: G20 coverage and gaps. *Clim. Policy* **22**, 158–174 (2022).
3. Creutzig, F. *et al.* Demand-side solutions to climate change mitigation consistent with high levels of well-being. *Nat. Clim. Chang.* **12**, 36–46 (2022).
